# Autonomic nervous system activity and the risk of nosocomial infection in critically ill patients with brain injury

**DOI:** 10.1186/s40635-020-00359-3

**Published:** 2020-11-25

**Authors:** Mathijs R. Wirtz, Jiri Moekotte, Kirsten Balvers, Marjolein M. Admiraal, Jean-Francois Pittet, Joe Colombo, Brant M. Wagener, J. Carel Goslings, Nicole Juffermans

**Affiliations:** 1 Department of Intensive Care Medicine, Onze Lieve Vrouwe Gasthuis and Amsterdam University Medical Centers, Amsterdam, The Netherlands; 2 Laboratory of Experimental Intensive Care and Anesthesiology of the Amsterdam University Medical Center, Amsterdam, The Netherlands; 3 Trauma Unit, Department of Surgery, Amsterdam University Medical Center, Amsterdam, The Netherlands; 4 Trauma Unit, Department of Surgery, Onze Lieve Vrouwe Gasthuis, Amsterdam, The Netherlands; 5 Department of Anesthesiology and Perioperative Medicine, University of Alabama at Birmingham, Birmingham, AL USA; 6 Department of Cardiology, Drexel University College of Medicine, and ANSAR Medical Technologies, Inc., Philadelphia, PA USA

**Keywords:** Autonomic nervous system, Brain injury, Immunosuppression, Nosocomial infection

## Abstract

**Purpose:**

Nosocomial infection contributes to adverse outcome after brain injury. This study investigates whether autonomic nervous system activity is associated with a decreased host immune response in patients following stroke or traumatic brain injury (TBI).

**Methods:**

A prospective study was performed in adult patients with TBI or stroke who were admitted to the Intensive Care Unit of our tertiary university hospital between 2013 and 2016. Heart rate variability (HRV) was recorded daily and assessed for autonomic nervous system activity. Outcomes were nosocomial infections and immunosuppression, which was assessed ex vivo using whole blood stimulations with plasma of patients with infections, matched non-infected patients and healthy controls.

**Results:**

Out of 64 brain injured patients, 23 (36%) developed an infection during their hospital stay. The ability of brain injured patients to generate a host response to the bacterial endotoxin lipopolysaccharides (LPS) was diminished compared to healthy controls (*p* < 0.001). Patients who developed an infection yielded significantly lower TNF-α values (86 vs 192 pg/mL, *p* = 0.030) and a trend towards higher IL-10 values (122 vs 84 pg/mL, *p* = 0.071) following ex vivo whole blood stimulations when compared to patients not developing an infection. This decreased host immune response was associated with altered admission HRV values. Brain injured patients who developed an infection showed increased normalized high-frequency power compared to patients not developing an infection (0.54 vs 0.36, *p* = 0.033), whereas normalized low-frequency power was lower in infected patients (0.46 vs 0.64, *p* = 0.033).

**Conclusion:**

Brain injured patients developing a nosocomial infection show parasympathetic predominance in the acute phase following brain injury, reflected by alterations in HRV, which parallels a decreased ability to generate an immune response to stimulation with LPS.

## Introduction

Stroke and traumatic brain injury (TBI) result in high rates of infectious complications, with subsequent high mortality rates [1, 2]. Patients admitted to the intensive care unit (ICU) are vulnerable to nosocomial infection, for example due to iatrogenic factors such as indwelling catheters and risk of aspiration prior to intubation [3]. However, these risk factors do not fully explain the disproportional higher infection rates in brain injured patients when compared to the general ICU population [1, 4, 5]. Brain injury-mediated immunosuppression is a recognized clinical phenomenon in brain injured patients [2, 4, 5].

Following neurotrauma, a disruption in the well-balanced interplay between the nervous and immune system occurs within hours of the insult and can last for several weeks [6]. Cerebral inflammatory responses produce pro-inflammatory cytokines and an influx of immune cells, causing neural cell death and brain damage [7]. Possibly, this initial inflammatory burst exhausts the immune system causing peripheral immunosuppression. The mechanism by which this occurs is not fully understood, but activation of the autonomic nervous system may contribute to immunosuppression following brain injury [7, 8]. A non-invasive method to investigate autonomic nervous system activity is through heart rate variability (HRV) analysis, describing interval changes between successive heartbeats [9]. Cardiac rhythm is regulated by the autonomic nervous system through alterations in sympathetic and parasympathetic tone resulting in changes in HRV. These alterations may be physiologic, but can also be the result of (critical) illness. HRV therefore represents a complex reflection of autonomic responsiveness of the cardiovascular system to changes in autonomic outflow and provides information on autonomic nervous system activity and sympathovagal balance. Increased parasympathetic nervous system activity correlates with immunosuppression and mortality in brain injured patients [10, 11]. This occurs through vagal nerve stimulation activating the cholinergic anti-inflammatory pathway, resulting in decreased systemic pro-inflammatory cytokine production, such as TNF-α [8].

This study aimed to elucidate the role of the autonomic nervous system in the risk of developing infections following brain injury. The primary aim of this study was to determine whether parasympathetic surges are associated with immunosuppression and nosocomial infection rates in brain injured patients. A secondary aim was to elucidate temporal trends in autonomic nervous system activity along with patterns of inflammatory response profiles associated with brain injury-mediated immunosuppression.

## Materials and methods

This prospective study was performed from March 2013 to July 2016 in our tertiary university hospital in the Netherlands. This study was approved by the ethics committee of the Amsterdam University Medical Centers. Patients were included following informed consent from next of kin and the study was conducted according to the principles of the Declaration of Helsinki and adhering to good clinical practice guidelines and the Medical Research Involving Human Subjects Act (WMO).

### Population

All patients ≥ 18 years old were eligible for inclusion if they suffered from TBI or stroke (ischemic or hemorrhagic) and required admission to the intensive care unit. Exclusion criteria included expected death within 1 h of admission, burn or inhalation injury or emergency thoracotomy or cardiopulmonary resuscitation with chest compressions before ICU arrival. Patients with known pregnancy, receiving immunosuppressive medication prior to admission, with a known do-not-resuscitate order and patients already enrolled in a concurrent ongoing interventional randomized clinical trial were also excluded.

### Heart rate variability recordings

Patients were hemodynamically monitored during their ICU or medium care stay. For HRV analysis, the heart rate of patients was recorded from the patient bedside monitor with specialized software from our hospital’s technical department (HeartRateMonitor3, Academic Medical Center, Amsterdam, The Netherlands). This software determines the position of the R peaks in the patient’s electrocardiography (ECG) at a sampling rate of 1000 Hz. Ten-minute epochs of the ECG were recorded directly after admission to the ICU and subsequently four times daily during the admission period. Daily recorded 10-min epochs were pooled together to calculate a daily mean to prevent circadian variation.

### Blood samples

Blood samples were collected on the admission day and 24 and 72 h thereafter (days 0, 1 and 3). The blood samples were centrifuged for 10 min at 1750 relative centrifugal force (RCF) at 18 °C. The upper two-thirds of the plasma was centrifuged again to obtain platelet-free plasma and stored as 250-µL aliquots at – 80 °C.

### Definition of infection

Infection criteria were adopted from the MARS project (Molecular Diagnosis and Risk Stratification of Sepsis) [12], which has provided criteria for infection classification that were modified from the Centers for Disease Control (CDC) and International Sepsis Forum (ISF) criteria. All available clinical, microbiological, and radiological evidence of infections was collected. Infections were scored based in their infection source and certainty of diagnosis, resulting in definite, probable and possible infections. The MARS criteria for infections can be found in the online Additional file 1, ‘criteria for diagnosed infections’.

### Antibiotic management

Selective digestive tract decontamination (SDD), consisting of parenteral and enteral antimicrobials, was administered to patients with an expected ICU stay of > 3 days. This includes a 4-day regime of intravenous cefotaxime. Also, an enteral nonabsorbable suspension containing polymyxin, tobramycin and amphotericin B was administered through a nasogastric tube four times a day. Orabase paste was applied four times a day to the oropharyngeal mucosa. Furthermore, antibiotics chosen based on local protocol and expert opinion were started empirically when an infection was suspected.

### Whole blood stimulation

Whole blood stimulations were performed as described before [13]. Briefly, heparin-anticoagulated blood from a healthy volunteer was collected. 500 µL whole blood samples were diluted in a 1:1 ratio with RPMI (Roswell Park Memorial Institute, Buffalo, USA), supplemented with glutamine 0.3 g/L and lipopolysaccharide (LPS *E. coli*; O111:B4 Ultrapure SIGMA, 1 ng/mL). Subsequently, 100 µL of plasma from either infected or non-infected patients was added. Whole blood stimulated with LPS without the addition of patient plasma served as a positive control. Whole blood buffered with RPMI without the addition of patient plasma or LPS served as a negative control. The samples were then incubated for 24 h at 5% CO_2_ at 37 °C. After incubation, the samples were centrifuged (1200 RCF at 18 °C for 10 min) and the upper two-thirds of the plasma was collected and stored at − 20 °C.

### ELISA

TNF-α and IL-10 levels were measured in the supernatant collected after the whole blood stimulations by enzyme-linked immunosorbent assays (ELISA) according to the manufacturer’s instructions (R&D Systems, Abingdon, United Kingdom).

### Outcome

The primary outcome of our study were nosocomial infections, autonomic nervous system activity and the extent of immunosuppression, defined as the outcome of ex vivo whole blood stimulations. Secondary outcomes included temporal trends in heart rate variability and immunosuppression, duration of ventilation, length of hospital and ICU stay and 28-day mortality.

### Statistical analyses

HRV recordings were analyzed with HRV algorithms in MATLAB software (MathWorks, Natick, USA) adhering to international standards [14]. The rationale behind the algorithms is described in more detail in the online Additional file 1. Normalized units of high-frequency power (HFnu) as a reflection of parasympathetic activity and normalized units of low-frequency power (LFnu) as a reflection of sympathetic activation were used to assess autonomic activity [14]. Also low-frequency:high-frequency (LF:HF) ratio was analyzed, as this ratio is considered to reflect either the sympathovagal balance or sympathetic nervous system activity [10].

All patients with definite or probable infections were selected for ex vivo whole blood stimulation tests. These patients were compared to non-infected patients, matched for age and APACHE II scores.

Distribution of variables was examined for normality using the Kolmogorov–Smirnov test. Continuous variables are expressed as mean and standard deviation (SD) or median and interquartile range (IQR) and were analyzed using the Student’s *t*-test or the Mann–Whitney U test, depending on the distribution. Categorical factors are expressed as proportions and were analyzed by Chi-square testing. Testing between infected patients, non-infected patients and controls in ex vivo whole blood stimulation tests was done with the one-way ANOVA test. *p *Values less than 0.05 were considered statistically significant.

## Results

Of 1712 screened patients, of these, 151 patients were eligible for inclusion, of whom 75 patients were excluded because no informed consent was obtained, they were expected to die within 1 h after admission, or they were discharged within 24 h of ICU admission. A total of 12 patients were missed for inclusion due to logistic difficulties. A total of 64 brain injured patients were included. Patient demographics and baseline characteristics are displayed in Table 1. Patients were 51 ± 19 years old and 73% were male. 50 patients (78%) suffered from TBI (with or without subsequent hemorrhagic stroke), 9 patients (14%) from ischemic stroke and 5 patients (8%) were admitted with hemorrhagic stroke. Median GCS was 7 on emergency department admission and patients were critically ill and severely injured, as reflected by a median APACHE II score of 19 and a mean ISS score of 24.

**Table 1:** Baseline characteristics of brain injured patients

	All patients (*n* = 64)	Infected (*n* = 23)	Non-infected (*n* = 41)
Main characteristics			
Age (years, *n*, %)	51 (19.3)	52 (18.2)	50 (20.1)
Male gender (*n*, %)	47 (73%)	15 (65%)	32 (78%)
Reason for ICU admission (*n*, %)			
TBI	50 (78%)	18 (78%)	32 (78%)
iCVA	9 (14%)	2 (9%)	7 (17%)
hCVA	5 (8%)	3 (13%)	2 (5%)
Admission injury/illness severity scores			
ISS (mean, SD)	24 (11)	26 (12)	23 (11)
AIS thorax ≥ 3 (*n*, %)	13 (26%)	5 (28%)	8 (25%)
AIS abdomen/pelvis ≥ 3 (*n*, %)	2 (4%)	1 (6%)	1 (3%)
AIS extremity ≥ 3 (*n*, %)	4 (8%)	0 (0%)	4 (13%)
AIS external ≥ 3 (*n*, %)	0 (0%)	0 (0%)	0 (0%)
GCS (median, IQR)	7 (3–9)	7 (3–8)	6 (3–9)
SAPS 2 (mean, SD)	43.7 (11.3)	45.5 (9.3)	42.7 (12.3)
APACHE II (median, IQR)	19 (16–23)	19 (17–21)	19 (15–23)
Comorbidity			
Prehospital beta-blocker use (*n*, %)	10 (16%)	4 (17%)	6 (15%)
Hypertension (*n*, %)	14 (22%)	5 (22%)	9 (22%)
CVA (*n*, %)	6 (9%)	3 (13%)	3 (7%)
Cardiac pathology (*n*, %)	11 (17%)	3 (13%)	8 (20%)
Diabetes mellitus (*n*, %)	6 (9%)	2 (9%)	4 (10%)
COPD (*n*, %)	4 (6%)	1 (4%)	3 (7%)
Alcohol abuse (*n*, %)	5 (8%)	2 (9%)	3 (7%)
Admission clinical parameters			
Heart rate (bpm, mean, SD)	80 (17)	83 (15)	79 (18)
SBP (mmHg, mean, SD)	132 (29)	132 (37)	133 (24)
Temperature (°C, mean, SD)	35.6 (1.2)	35.5 (1.3)	35.7 (1.2)
Admission lab parameters			
Hb (mmol/l, median, IQR)	7.9 (6.7–8.5)	7.2 (6.3–8.2)*	8.0 (7.5–8.8)
WCC (× 10^9/l, mean SD)	11.8 (4.7)	11.6 (5.8)	11.9 (4.0)
CRP (mg/l, median, IQR)	3.8 (0.7–6.7)	3.8 (2–7.4)	4.9 (0.6–7.8)

Data are presented as mean standard deviation, median and interquartile range or absolute number and percentage

*ICU* intensive care unit, *TBI* traumatic brain injury, *iCVA* ischemic cerebrovascular accident, *hCVA* hemorrhagic cerebrovascular accident, *ISS* Injury Severity Score, *AIS* Abbreviated Injury Scale, *GCS* Glasgow Coma Score, *SOFA* Sequential Organ Failure Assessment Score, *SAPS 2* Simplified Acute Physiology Score 2, *APACHE II* Acute Physiology and Chronic Health Evaluation 2 score, *MI* myocardial infarction, *AF* atrial fibrillation, *COPD* chronic obstructive pulmonary disease, *SBP* systolic blood pressure, *Hb* hemoglobin, *WCC* white cell count, *CRP* C-reactive protein

*Significantly different from non-infected group

^†^Cardiac pathology includes MI and AF, as well as cardiac valve pathology and other (paroxysmal) rhythm disorders

### Infections

**Fig. 1:**
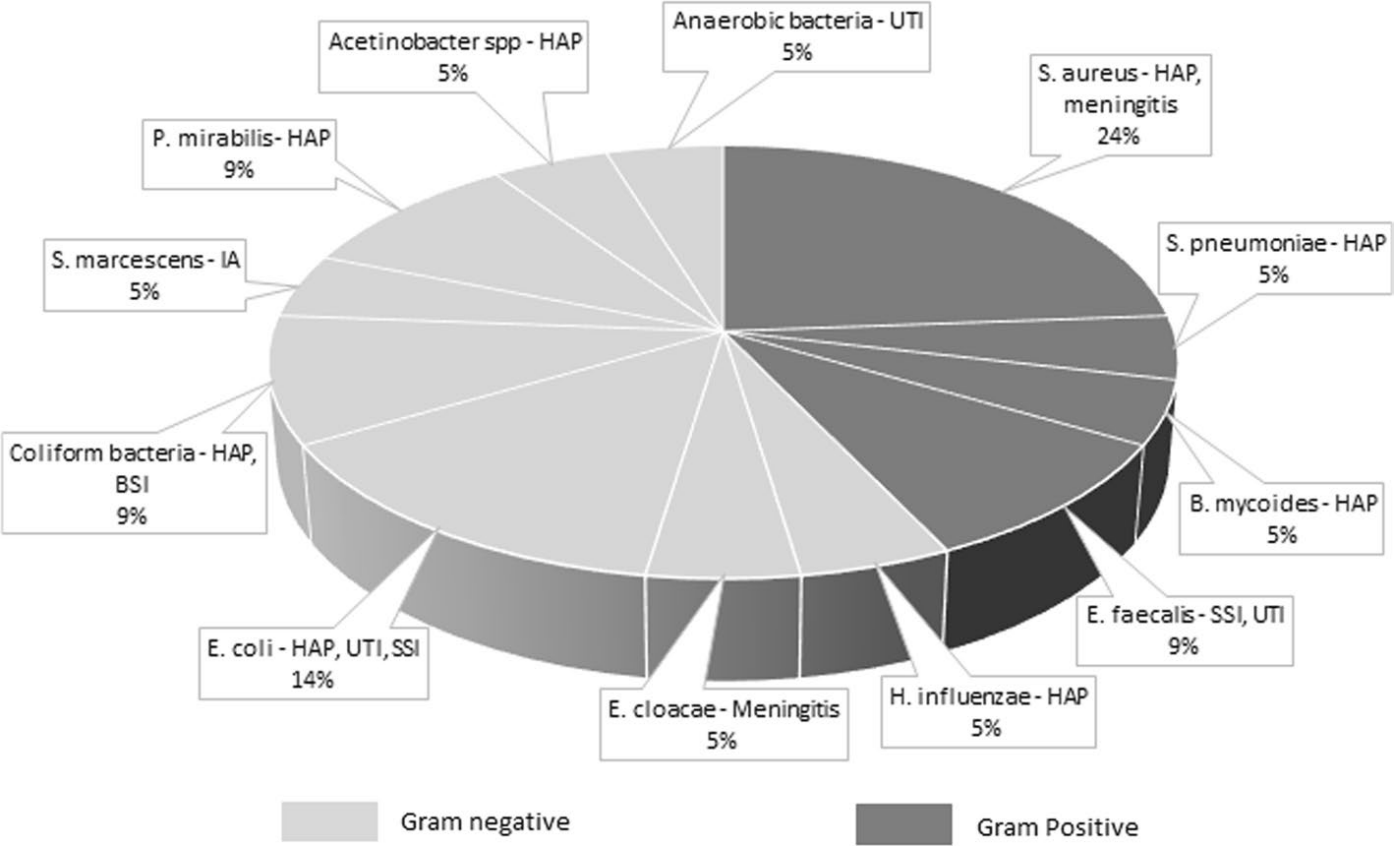
Pathogens found in positive microbiology cultures.* HAP* hospital acquired pneumonia,* SSI* surgical site infection,* UTI* urinary tract infection,* BSI* blood stream infection,* IA* intracranial abscess

Baseline characteristics between patients who did and did not develop an infection were similar, with the exception of hemoglobin levels being lower in infected patients (Table 1). The type and probability of infections among included patients are presented in Additional file 1: Table 1. A total of 23 patients (36%) developed at least 1 infection during their hospital stay. Infections in 8 patients were classified as definite, in 5 patients as probable and in 10 as possible. The total number of infections was 28, as 4 patients developed multiple infections. Pneumonia was most frequently observed (*n* = 16), followed by urinary tract infections (*n* = 4) and intracranial infections (*n* = 4). Other observed infections include primary blood stream infections and surgical site infections. Two-thirds of infected patients developed an infection during their initial ICU stay, while the others developed an infection after transfer to the ward. *Staphylococcus aureus* was most frequently cultured (25%, Fig. 1). Patients who developed an infection did so after a median of 6 days (IQR 3–12) after admission. More invasive procedures were performed in infected patients compared to patients not developing an infection (see Table 2). No difference was seen in the use or duration of antibiotic treatment. Prehospital use of beta-blockers was also similar (15 vs 15%, *p* = 0.80), as was its use at the day of admission to the ICU (5 vs 9%, *p* = 0.55).

**Table 2:** Treatment on ICU and outcome

	All patients (*n* = 64)	Infected (*n* = 23)	Non-infected (*n* = 41)	*p*-value
Invasive procedures				
Surgery (*n*, %)	38 (59)	17 (74)	21 (51)	0.076
Amount of surgical procedures (*n*, median, IQR)	1 (0–1)	1 (0–3)	1 (0–1)	0.006
Tracheostomy (*n*, %)	10 (16)	8 (35)	2 (5)	0.002
Intracranial device (*n*, %)	20 (31)	12 (52)	8 (20)	0.007
Duration of Intracranial device (days, median, IQR)	0 (0–2)	1 (0–6)	0 (0–0)	0.005
Central venous catheter (*n*, %)	26 (41)	12 (52)	14 (34)	0.159
Duration of central venous catheter (days, median, IQR)	0 (0–5)	2 (0–8)	0 (0–3)	0.103
Treatment during ICU/CMC stay				
Antibiotics (*n*, %)	48 (75)	19 (83)	29 (71)	0.292
SDD (*n*, %)	44 (69)	18 (78)	26 (63)	0.219
Other than SDD (*n*, %)	37 (58)	16 (70)	21 (51)	0.154
Duration of antibiotics (days, median, IQR)	5 (1–7)	5 (2–9)	4 (0–7)	0.126
Vasopressor (*n*, %)	35 (55)	17 (74)	18 (44)	0.021
Duration of vasopressor use (days, median, IQR)	1 (0–2)	1 (0–5)	0 (0–2)	0.016
Beta-blocker (*n*, %)	12 (19)	7 (30)	5 (12)	0.073
Duration of beta-blocker use (days, median, IQR)	0 (0–0)	0 (0–2)	0 (0–0)	0.066
Mechanically ventilated (*n*, %)	61 (95)	23 (100)	38 (93)	0.184
Duration of ventilation (days, median, IQR)	3 (2–7)	6 (2–11)	2 (2–5)	0.042
Outcome				
Onset of (first) infection (days, median, IQR)	NA	6 (3–12)	NA	NA
ICU stay (days, median, IQR)	4.5 (3–9)	8 (3–14)	4 (2–6.5)	0.011
Total length of hospital stay (days, median, IQR)	12 (6–26)	24 (10–44)	9 (5–17)	0.001
Discharge location (*n*, %)				
Home	20 (31)	4 (17)	16 (39)	0.073
Another hospital	17 (27)	8 (35)	10 (24)	0.375
Nursing home	14 (22)	7 (30)	7 (17)	0.215
28-day mortality (*n*, %)				
All causes	12 (19)	4 (17)	8 (19)	0.835
Brain injury	10 (8)	3 (13)	7 (16)	0.670
MOF	2 (3)	1 (4)	1 (2)	0.674

Data are presented as median and interquartile range or absolute number and percentage

*SDD* selective decontamination of the digestive tract, *ICU* intensive care unit

### Cytokine production after whole blood stimulation

**Fig. 2:**
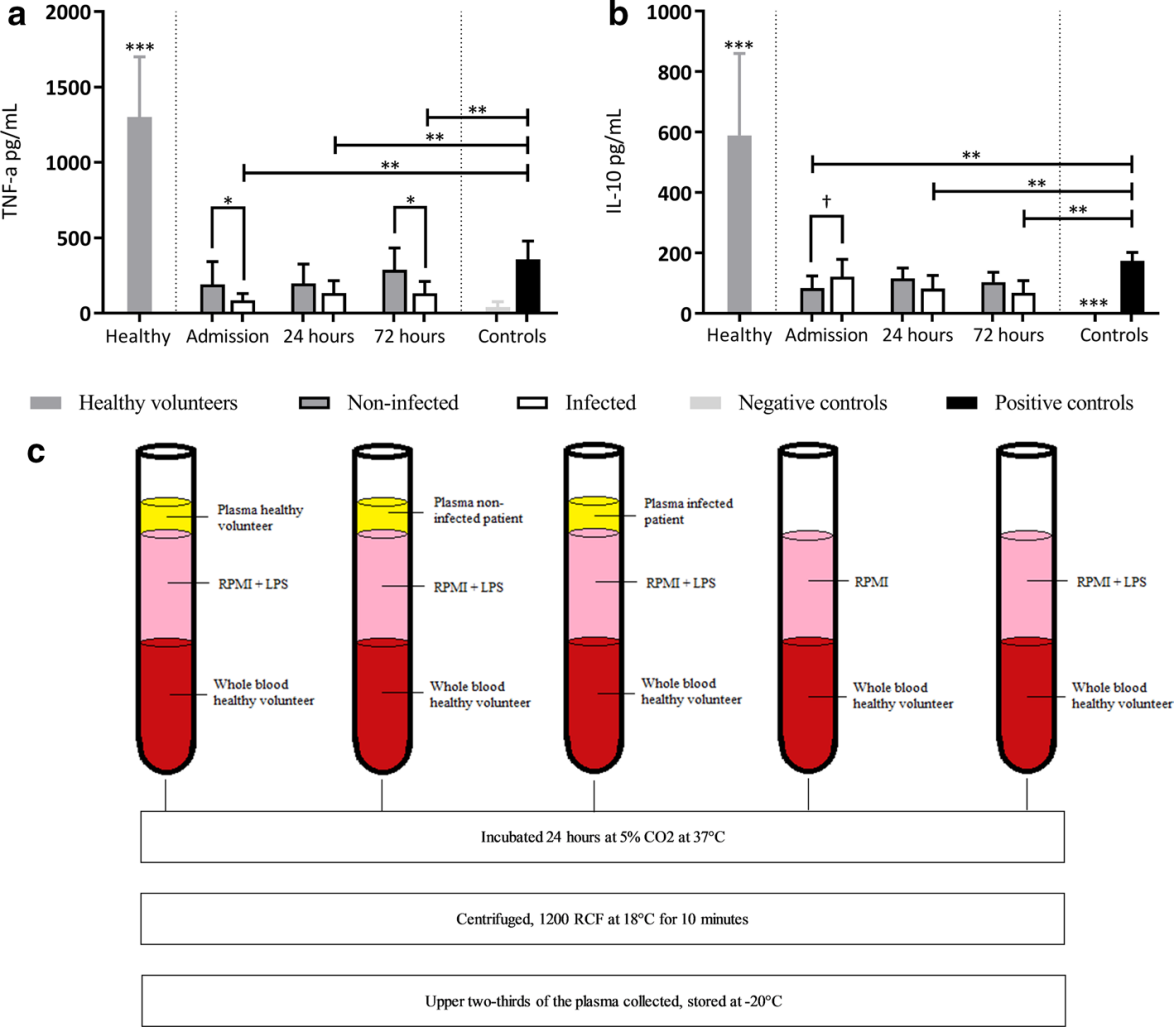
Cytokine production after ex vivo whole blood stimulation. Cytokine production after whole blood stimulation with LPS for samples collected on admission, and 1 and 3 days after admission to the ICU. Values are represented as mean and standard deviation. **a** TNF-α production measured after 24 h of incubation time. **b** IL-10 production measured after 24 h of incubation time. **c** Experimental set-up of whole blood stimulations. Healthy = whole blood stimulations with LPS and incubated with plasma samples of healthy volunteers. Non-infected = whole blood stimulations with LPS incubated with plasma samples of patients without an infection. Infected = whole blood stimulations with LPS incubated with plasma samples of patients with a probable or definite infection. Negative controls = whole blood buffered with RPMI without LPS and without the addition of patient plasma. Positive control = whole blood stimulation with LPS without the addition of patient plasma. †p < 0.10; *p < 0.05; **p < 0.01; ***p < 0.001 compared to all other measurements

Cytokine production in response to ex vivo stimulation with LPS was measured in 12 patients with definite or probable infections and compared to non-infected matched controls as well as healthy controls (see Fig. 2). Brain injured patients had a significant lower ability to generate TNF-α and IL-10 production when compared to healthy controls (all *p* < 0.001). On admission to the ICU, patients who developed an infection during their hospital stay had lower TNF-α production in response to LPS compared to non-infected patients (86 vs 192 pg/mL, *p* = 0.030). This difference was also apparent three days after admission (113 vs 287 pg/mL, *p* = 0.024).

Compared to patients without an infection, a trend towards higher IL-10 values at baseline was found in infected patients (122 vs 84 pg/mL, *p* = 0.071).

### Heart rate variability

Results of heart rate variability analysis of the acute phase following admission after brain injury are shown in Table 3. At ICU admission, patients who acquired an infection later during their hospital stay had significantly lower normalized low-frequency power (LFnu, 0.46 vs 0.64, *p* = 0.033) accompanied with higher normalized high-frequency power (HFnu, 0.54 vs 0.36, *p* = 0.033) when compared to patients who did not acquire an infection. Furthermore, a trend was found towards a lower LF:HF ratio in infected patients compared to non-infected patients (0.93 vs 1.82, *p* = 0.065).

**Table 3:** Admission HRV parameters

HRV parameter	Infected (*n* = 9)	Not infected (*n* = 19)	*p*-value
LF (msec^2^, median, IQR)	53 (5–121)	117 (58–339)	0.029
HF (msec^2^, median, IQR)	72 (27–90)	72 (22–177)	0.572
TP (msec^2^, median, IQR)	349 (97–750)	353 (247–1218)	0.446
LF:HF ratio (median, IQR)	0.93 (0.43–2.45)	1.82 (1.33–4.36)	0.065
LFnu (mean, SD)	0.46 (0.24)	0.64 (0.18)	0.033
HFnu (mean, SD)	0.54 (0.24)	0.36 (0.18)	0.033

Data are presented as mean and SD or as median and IQR

*LF* low frequency, *HF* high frequency, *TP* total power, *LF:HF ratio* ratio between low and high frequency, *LFnu* normalized units of low-frequency power, *HFnu* normalized units of high-frequency power

**Fig. 3:**
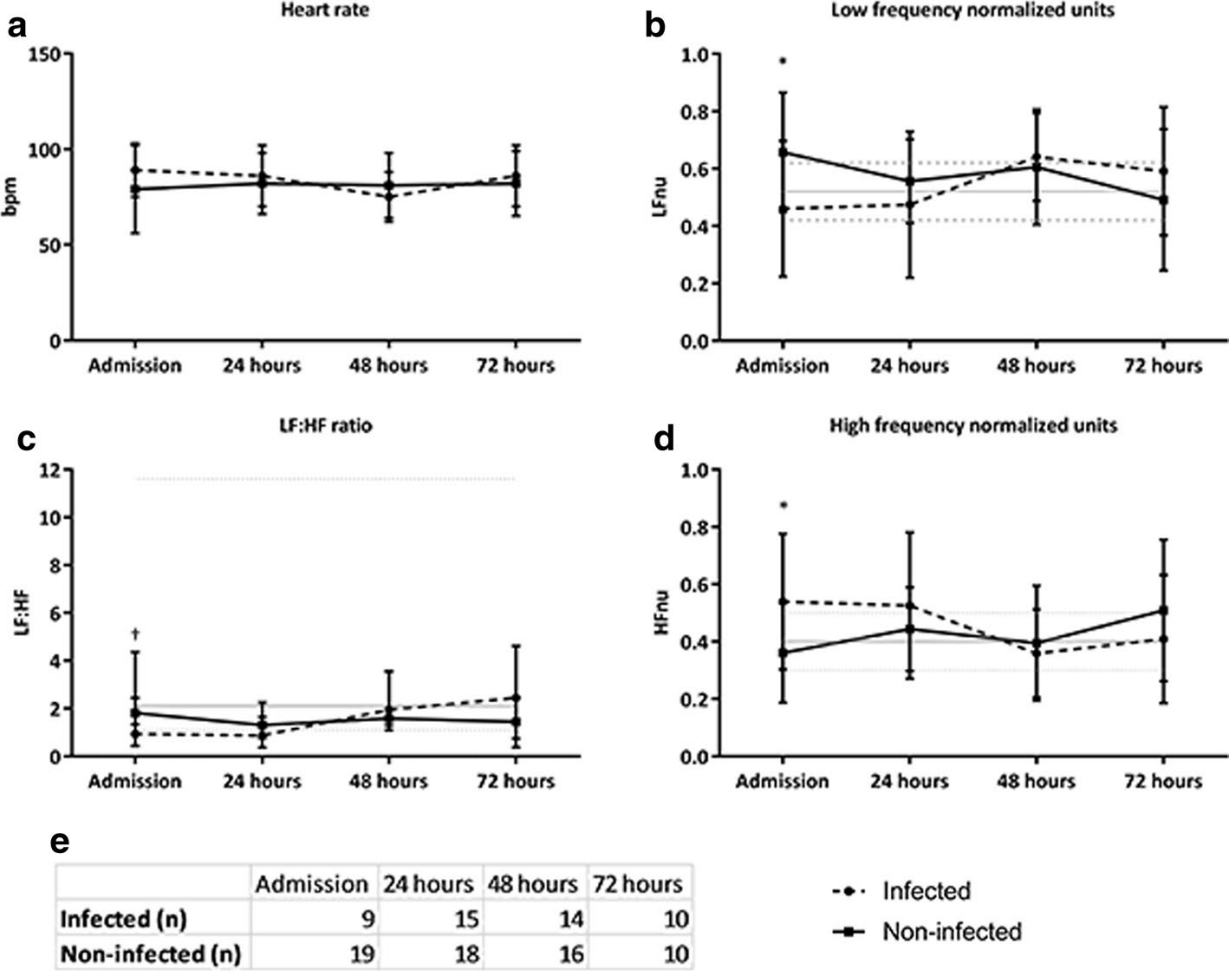
Longitudinal trends of heart rate variability. Daily averages of heart rate variability parameters in infected (black-dotted lines) and non-infected patients (black lines). Grey and dotted lines represent normal values in healthy volunteers as previously described in the literature52 (in figure **c** median and range and in figure **b** and **d** mean and standard deviation). **a** Heart rate in beats per minute presented as mean and standard deviation. **b** Low frequency normalized units (LFnu) presented as mean and standard deviation. **c** LF:HF ratio presented as median and interquartile range. **d** High frequency normalized units (HFnu) presented as mean and standard deviation. **e** Number of patients included for every time point. †*p* < 0.10; *p < 0.05

Figure 3 shows the longitudinal patterns of HRV after brain injury. Following the acute phase, patients not developing an infection showed a decrease in LFnu and LF:HF and an increase in HFnu in the first 24 h, while these values remained stable in patients who developed an infection. After 24 h, there was no significant difference between the groups. Patients developing an infection during their stay in the hospital had a median LF:HF ratio below reference values of healthy volunteers, as well as a higher HFnu in the first 24 h after admission to the ICU. Patients without an infection showed values within these reference ranges.

### Outcome

Duration of mechanical ventilation was significantly longer in patients who developed an infection compared to those who did not (6 vs 2 days, *p* = 0.042). More often, these patients required the use of vasopressors (74 vs 44%, *p* = 0.021) and required these for a longer time period (1 vs 0 day, *p* = 0.016). Also, hospital length of stay was significantly prolonged when patients developed an infection (24 vs 9 days, *p* = 0.001), as was ICU stay (8 vs 4 days, *p* = 0.011). However, 28-day mortality rates did not differ between infected and non-infected patients (17 vs 19%, *p* = 0.835, see Table 2).

## Discussion

Out of 64 brain injured patients, 36% developed an infection. All brain injured patients showed a decreased ability to generate an immune response to bacterial LPS stimulation ex vivo compared to healthy controls. When compared to non-infected patients, patients developing an infection had a reduced immunoresponsiveness, reflected by decreased TNF-α values after ex vivo bacterial stimulation. The development of an infection was associated with a parasympathetic surge at ICU admission. At 72 h after injury, parasympathetic activity decreased, whereas TNF-α production in response to a bacterial stimulus remained abrogated.

Despite the fact that 75% of patients received antibiotic treatment during their ICU stay, with 53% already starting on admission, 36% of patients developed an infection during their hospital stay. This incidence is similar to findings in previous studies [1, 2]. Of note, most pathogens found in positive microbiology cultures in infected patients in our study were covered by SDD prophylaxis. Also, the number of patients receiving antibiotic treatment did not differ between patients developing an infection and those who did not, even after correcting for the use of SDD. This shows that antibiotic (prophylactic) treatment may not effectively prevent infections in these patients. In brain injury patients, evidence for the use of prophylactic antibiotics is scarce and somewhat conflicting, with most studies showing no effect of prophylactic antibiotics in preventing pneumonia [4, 15].

Immunosuppression following brain injury is a phenomenon that is increasingly recognized. Our study shows that reduced immunoresponsiveness increases susceptibility to post-injury infection. The fact that a reduced immunoresponsiveness is apparent directly after injury suggests that it is a cause and not the result of an infection. This is supported by a recent study in which an immunosuppressive phenotype has been found in lung tissue of brain injured mice that were inoculated with Streptococcus pneumonia, even when mice were inoculated two months after the primary injury [16]. This stresses the fact that immunosuppression following brain injury is a severe complication with long lasting consequences.

Patients developing an infection in our study required more and longer supportive care and stayed in the ICU longer. However, as patients showed a parasympathetic surge along with immunosuppression on admittance to the ICU, we believe these procedures are a consequence of the infection, rather than the cause. This is underlined by the fact that 77% of procedures were performed after the infection was diagnosed.

Significant autonomic regulation dysfunction in brain injured patients has been reported, with lower levels of overall HRV when compared to healthy controls [10, 17]. Our data demonstrate an autonomic shift towards parasympathetic dominance preceding the occurrence of infection, reflected by higher HFnu values. This parasympathetic surge corroborated with a systemic immune suppression as reflected by a decreased ability to generate TNF-α in ex vivo stimulated plasma samples. Previously published data confirmed that parasympathetic activity correlated strongly with lower plasma TNF-α levels [10]. Thereby parasympathetic activity may up-regulate the brain’s immune system at the expense of the body’s immune system, as was shown in septic trauma patients [18]. Also, it has been shown that brain injury leads to increased activity of vagal nerve parasympathetic fibers, which in turn leads to downregulation of TNF-α production through the cholinergic pathway without affecting IL-10 production [19]. What is more, a study evaluating HRV in the acute phase after ischemic stroke found that increased vagal activity (increased HFnu) was predictive of the development of subacute infections [20].

Our findings may have potential clinical consequences. HRV, in particular HFnu, may distinguish patients at risk of acquiring an infection and serve as an indicator to initiate therapy. Several potential therapies intervening in autonomic regulation have been described. Trials on counteracting autonomic nervous system activity by beta-blockade show conflicting results [21–23]. A recent systematic review in TBI patients showed that infections occurred more often in patients receiving beta-blockade [24]. In our study, total in-hospital administration of beta-blockers was similar between infected and non-infected patients, although a trend towards more and longer beta-blocker use was found in patients who developed an infection. The use of beta-blocking agents may result in higher parasympathetic activity, as measured by HRV [25]. In our study parasympathetic hyperactivity preceded development of an infection and was not related to beta-blocker administration. Thereby, our results do not support the use of beta-blockers in brain injured patients already in a parasympathetic hyperactive state at admission to the ICU.

Several therapies targeting the cholinergic pathway have also been suggested in brain injury patients [26]. Research with animal models demonstrated that inhibition of cholinergic signaling, by vagotomy or by using acetylcholine receptor-deficient mice, enabled reversal of the observed immune hyporesponsiveness and prevented pneumonia after stroke [27]. Preclinical studies investigating the administration of muscarinic- and nicotinic-acetylcholine receptor antagonists showed a benefit in neural survival by preventing the effects of acute elevation of acetylcholine in parasympathetic hyperactivation. What is more, pharmacological inhibition of the nicotinic-acetylcholine receptor reduced lung injury and mortality in mice with a post-stroke pneumonia [28]. However, the effect of these agents needs further research, specifically focusing on the effect of these agents on the parasympathetic surge in the acute phase after brain injury and the relation with immune functioning and infection rates.

## Limitations

We did not retain raw electrocardiography data, nor did we record respiratory variability, rendering it more difficult to isolate the parasympathetic activity signal from the sympathetic activity signal [29]. Also, only explorative analysis could be performed without correcting for multiple comparisons given our small cohort. For the same reason no subgroup analysis of TBI and stroke patients could be performed. Therefore, heterogeneity may have influenced our results. Additional replication studies using larger cohorts of patients will be necessary to confirm our findings. Furthermore, uniform infection criteria were used in this study, however some patients may have received an unjustified diagnosis of infection, especially patients in the possible infection category. Lastly, the use of HRV has some drawbacks. Due to the large heterogeneity and poor description of the methodology of studies using HRV analysis and the lack of a uniform protocol for its effectuation, comparisons with contemporary literature may be hampered.

## Conclusion

Infection occurs frequently after brain injury. Hyperactivity of the parasympathetic nervous system in the acute phase after injury may predispose patients to the development of an infection, due to a decreased systemic immunoresponsiveness. Whether high parasympathetic outflow, as reflected by the corresponding HRV indices, might thus function as an early indicator of patients at risk for infection needs further investigation. Results of this study may stimulate research aiming at improving the autonomic balance in order to reduce infection rates.

## Supplementary information

**Additional file 1:** Criteria for diagnosed infections.

## Data Availability

The data that support the findings of this study are available from the corresponding author, MW, upon reasonable request.
