# Supplementary material for: Autonomic nervous system activity and the risk of nosocomial infection in critically ill patients with brain injury
Source: Intensive Care Med Exp. 2020 Nov 25;8:69. doi: 10.1186/s40635-020-00359-3 (PMC7688871; doi:10.1186/s40635-020-00359-3)
Supplement: Supplementary file 1 — Additional file 1: Criteria for diagnosed infections. [file 40635_2020_359_MOESM1_ESM.docx]

# Additional file

# Criteria for diagnosed infections

| **Hospital Acquired Pneumonia** | |
| --- | --- |
| **Clinical setting** | Patients with respiratory symptoms that started more than 48 hours after hospital admission, but without mechanical ventilation (or onset of pneumonia within 48 hours after start of mechanical ventilation). |
| **Possible** | 1. Abnormal chest radiograph of uncertain cause  **and** low clinical suspicion of pneumonia with at least one of the following symptoms/signs:   1. Cough; 2. New onset of purulent sputum of change in character of sputum; 3. Fever or hypothermia; 4. Leukocytosis; 5. Elevated CRP (>30mg/L); 6. Hypoxemia (pO2<60mmHg). |
| **Probable** | 1. Evident new or progressive radiographic infiltrate, consolidation, cavitation, or pleural  effusion  **and** high clinical suspicion of pneumonia with at least two of the criteria at possible  **and** one or more of the following:   1. Isolation of an etiology agent from a specimen obtained by transtracheal aspirate, bronchial brushing, or biopsy; 2. Likely/possible respiratory pathogen in concentrations below threshold (10^4^ in BAL and 10^3^ in protected specimen brush) using quantitative cultures of a lower respiratory tract sample (endotracheal aspirate, BAL, of protected specimen brush). |
| **Definite** | 1. Evident new or progressive radiographic infiltrate, consolidation, cavitation, or pleural  effusion  **and** high clinical suspicion of pneumonia with at least two of the criteria at probable  **and** at least one of the following:   1. Likely/possible respiratory pathogen cultured from blood; 2. Likely/possible respiratory pathogen in concentrations above threshold (10^4^ in BAL and 10^3^ in protected specimen brush) using quantitative cultures of a lower respiratory tract sample (endotracheal aspirate, BAL, of protected specimen brush); 3. Isolation of virus from or detection of viral antigen in respiratory secretions; 4. Diagnostic single antibody titer (IgM) of fourfold increase in paired sera (IgG) for pathogen; 5. Histopathologic evidence of pneumonia. |
| **Comment** | Sputum cultures are not useful in the diagnosis of pneumonia but may help identify the etiologic agent and provide antimicrobial susceptibility data. |

| **Intracranial infections: abscess** | |
| --- | --- |
| **Possible** | 1. At least two of the following signs of symptoms with no other recognized cause:   1. Headache; 2. Fever (>38°C); 3. Localizing neurologic signs; 4. Changing level of consciousness; 5. Confusion. |
| **Probable** | 1. See criteria ‘possible’  **and** at least one of the following:   1. Organisms seen on microscopic examination of brain or abscess tissue obtained by needle aspiration or by biopsy during a surgical operation or autopsy; 2. Radiographic evidence of infection, e.g. abnormal findings on ultrasound, CT scan, magnetic resonance imaging (MRI), or anteriogram |
| **Definite** | 1. Organisms cultured from brain tissue or dura; 2. Abscess or evidence of intracranial infection seen during a surgical operation of histopathologic examination. |

| **Community Acquired Pneumonia** | |
| --- | --- |
| **Clinical setting** | Patients with respiratory symptoms within 48 hours of hospital admission |
| **Possible** | 1. Abnormal chest radiograph of uncertain cause  **and** low clinical suspicion of pneumonia with at least one of the following symptoms/signs:   1. Cough; 2. New onset of purulent sputum of change in character of sputum; 3. Fever or hypothermia; 4. Leukocytosis; 5. Elevated CRP (>30mg/L); 6. Hypoxemia (pO2<60mmHg). |
| **Probable** | 1. Evident new or progressive radiographic infiltrate, consolidation, cavitation, or pleural  effusion  **and** high clinical suspicion of pneumonia with at least two of the criteria at possible  **and** one or more of the following:   1. Rales or dullness to percussion on physical examination of the chest 2. Positive rapid diagnostic test such as Legionella or pneumococcal |
| **Definite** | 1. Evident new or progressive radiographic infiltrate, consolidation, cavitation, or pleural  effusion  **and** high clinical suspicion of pneumonia with at least two of the criteria at probable  **and** isolation of a likely pulmonary pathogen, with at least one of the following  symptoms/signs:   1. Pathogen cultured from blood; 2. Pathogen in high concentration from a quantitative lower respiratory tract sample; 3. Isolation of virus from or detection of viral antigen in respiratory secretions; 4. Diagnostic single antibody titer (IgM) of fourfold increase in paired sera (IgG) for pathogen; 5. Histopathologic evidence of pneumonia. |
| **Comment** | Sputum cultures are not useful in the diagnosis of pneumonia but may help identify the etiologic agent and provide antimicrobial susceptibility data. |

| **Secondary meningitis** | |
| --- | --- |
| **Clinical setting** | Patients presenting with symptoms of meningitis up to 1 year after neurotrauma, neurosurgery, ENT-surgery, external ventricular drain, external lumbar drain, or ventriculo-peritoneal drain if there is a suspected infection in the peritoneal part |
| **Possible** | 1. At least two of the following signs of symptoms with no other recognized cause:   1. Fever (>38°C); 2. Headache; 3. Stiff neck; 4. Meningeal signs; 5. Cranial nerve signs; 6. Changing level of consciousness. |
| **Probable** | 1. See criteria ‘possible’  **and** at least one of the following:   1. Positive urine antigen test (pneumococcal); 2. Organisms cultured from blood. |
| **Definite** | 1. Organisms cultured from CSF (if low numbers of skin contaminants, then take chemical and clinical signs into account); 2. See criteria ‘possible’   **and** increased elevated protein, and/or decreased glucose in CSF (if bloody then positive is when leukocyte:erythrocyte ratio is >1:100; if not bloody then positive when leukocytes >100x10^6^/L)  **and** organisms seen on Gram stain of CSF |

| **Urosepsis in noncatheterized patients** | |
| --- | --- |
| **Possible** | 1. At least two of the following signs of symptoms with no other recognized cause:   1. Fever (>38°C); 2. Urgency; 3. Frequency; 4. Dysuria; 5. Pyuria. |
| **Probable** | 1. See criteria ‘possible’  **and** at least one of the following:   1. Positive Dipstick for leukocyte esterase and/or nitrate; 2. Pyuria (>10 white blood cells/mm^3^ or >3 white blood cells/high-power filed of unspun urine); 3. Organisms seen in Gram stain of unspun urine; 4. Frank pus expressed around urinary catheter; 5. At least two urine cultures with repeated isolation of the same uropathogen with ≥10^2^ colonies/mL in nonvoided specimens; 6. Urine culture with ≤10^5^ colonies/mL of a single uropathogen in a patient being treated with appropriate antimicrobial therapy; 7. Radiographic evidence of infection (e.g. ultrasound, computed tomography, magnetic resonance imaging, radiolabeled scan) |
| **Definite** | 1. See criteria ‘possible’  **and** urine culture with >10^5^ colonies/mL with no more than two species of microorganisms  2. Abscess or other evidence of infection seen on direct examination, during surgery, or by  histopathologic examination. |

| **Surgical site infections: deep wounds** | |
| --- | --- |
| **Clinical setting** | Patients presenting with symptoms or signs of wound infection within 30 days following surgery or trauma |
| **Possible** | 1. Infection that arises within 30 days of an operative procedure and at the site of surgical  intervention of within 1 year after implant placement:  **and** infection involves the fascia or muscle layers  **and** at least two of the following:   1. Pain or tenderness; 2. Localized swelling; 3. Redness; 4. Heat. |
| **Probable** | N.A. |
| **Definite** | 1. See criteria ‘possible’  **and** at least one of the following:   1. Purulent discharge from incision or drain; 2. Abscess seen during observation, (re)surgery, hostopathologic or radiographic examination; 3. Organisms cultured from tissue or drainage of affected site. |

| **Bloodstream infection (BSI): primary BSI** | |
| --- | --- |
| **Clinical setting** | Bloodstream infection in a patient without an evident focus |
| **Possible** | N.A. |
| **Probable** | N.A. |
| **Definite** | 1. At least one of the following:   1. Patient has a recognized pathogen (defined as a microorganism not usually regarded as a common skin contaminant, i.e. diphtheroids, Bacillus species, Propoinibacterium species, coagulase-negative staphylococci, or micrococci) cultured from two or more blood cultures 2. A common skin contaminant, i.e. diphtheroids, Bacillus species, Propoinibacterium species, coagulase-negative staphylococci, or micrococci) cultured from two or more blood cultures on separate occasions (including one drawn by venipuncture)   **and** the organism cultured from blood is not related to an infection at another site, including intravascular-access devices. |

# Artifact correction

The importance of a careful approach of artefact detection is emphasized by the fact that only a few artefacts present in a recording can already considerably disturb the results of heart rate variability analysis^1^. Therefore, a minimum epoch duration of 5 minutes was required of the heart rate recordings, shorter recordings were not eligible for analysis. Recordings with more than 10% artefacts were also discarded from further analysis. Artefacts were defined by an algorithm complying with the following criteria:

1. Interbeat intervals shorter than 250 milliseconds or greater than 2000 milliseconds (representing a heart rate below 30 or over 240 beats per minute).
2. Intervals differing more than 3 times the mean absolute deviation of 20 surrounding intervals.
3. The third artefact criterion is called the MAD/MED criterion, which described in detail in the original paper^2^. Briefly, the artefact detection threshold is placed midway between the minimal artefact difference (MAD) and the maximum expected difference in veridical beats (MED). Outliers are flagged as potential artifact.

Interbeat intervals were removed from the recordings when they were marked as an artefact by the algorithm. The algorithm then returned to the last pre-artefactual veridical beat and continued the analysis to ensure that subsequent artefacts were not missed. After running the algorithm, the entire course of raw data with plotted detected artefacts was visually inspected to ensure all marked artefacts were in fact artefacts. The best segment of each recording was then visually selected and the algorithm was used a second time for removal of encountered artefacts in these segments (with improved artefact thresholds due to a lower proportion of outliers). The selected fragments were analyzed with a moving window of 5 minutes. The window containing the fewest artefacts was used for heart rate variability analysis. Standard parameters of frequency domain methods were calculated^3^. Furthermore, outliers after power analyses were removed according to the Tukey method, removing values higher or lower than the upper or lower quartile 1.5 times the interquartile range^4^.

# References

1. Peltola, M.A. (2012). Role of editing of R-R intervals in the analysis of heart rate variability. Frontiers in physiology 3, 148.

2. Berntson, G.G., Quigley, K.S., Jang, J.F. and Boysen, S.T. (1990). An approach to artifact identification: application to heart period data. Psychophysiology 27, 586-598.

3. (1996). Heart rate variability: standards of measurement, physiological interpretation and clinical use. Task Force of the European Society of Cardiology and the North American Society of Pacing and Electrophysiology. Circulation 93, 1043-1065.

4. Ramsay, T. and Elkum, N. (2005). A comparison of four different methods for outlier detection in bioequivalence studies. Journal of biopharmaceutical statistics 15, 43-52.

| **Table 1. Type and distribution of infections** | | | | |
| --- | --- | --- | --- | --- |
| **Infection type** | **Infection probability** | | | |
|  | Definite | Probable | Possible | Total |
| Pneumonia | 0 | 6 | 10 | 16 |
| Hospital acquired pneumonia | 0 | 6 | 8 | 14 |
| Community acquired pneumonia | 0 | 0 | 2 | 2 |
| Urinary tract infection* | 0 | 1 | 3 | 4 |
| Intracranial infection | 4 | 0 | 0 | 4 |
| Meningitis | 3 | 0 | 0 | 3 |
| Intracranial abscess | 1 | 0 | 0 | 1 |
| Blood stream infection | 2 | 0 | 0 | 2 |
| Surgical site infection** | 2 | 0 | 0 | 2 |
| Total | 8 | 7 | 13 | 28 |
| ***Urinary tract infections were all classified as possible urosepsis in catheterized patients.**  ****Surgical site infections were all classified as deep surgical site infections.** | | | | |
